# APEH Inhibition Affects Osteosarcoma Cell Viability via Downregulation of the Proteasome

**DOI:** 10.3390/ijms17101614

**Published:** 2016-09-23

**Authors:** Rosanna Palumbo, Marta Gogliettino, Ennio Cocca, Roberta Iannitti, Annamaria Sandomenico, Menotti Ruvo, Marco Balestrieri, Mosè Rossi, Gianna Palmieri

**Affiliations:** 1 Institute of Biostructure and Bioimaging, National Research Council (CNR-IBB), Napoli 80134, Italy; rosanna.palumbo@cnr.it (R.P.); robertaiannitti@gmail.com (R.I.); annamaria.sandomenico@gmail.com (A.S.); menotti.ruvo@unina.it (M.R.); 2 Institute of Biosciences and BioResources, National Research Council (CNR-IBBR), Napoli 80131, Italy; ennio.cocca@ibbr.cnr.it (E.C.); marco.balestrieri@ibbr.cnr.it (M.B.); mose.rossi@ibbr.cnr.it (M.R.)

**Keywords:** acylpeptide hydrolase (APEH), proteasome, osteosarcoma cell lines, peptide inhibitor, anti-tumoral target

## Abstract

The proteasome is a multienzymatic complex that controls the half-life of the majority of intracellular proteins, including those involved in apoptosis and cell-cycle progression. Recently, proteasome inhibition has been shown to be an effective anticancer strategy, although its downregulation is often accompanied by severe undesired side effects. We previously reported that the inhibition of acylpeptide hydrolase (APEH) by the peptide SsCEI 4 can significantly affect the proteasome activity in A375 melanoma or Caco-2 adenocarcinoma cell lines, thus shedding new light on therapeutic strategies based on downstream regulation of proteasome functions. In this work, we investigated the functional correlation between APEH and proteasome in a panel of cancer cell lines, and evaluated the cell proliferation upon SsCEI 4-treatments. Results revealed that SsCEI 4 triggered a proliferative arrest specifically in osteosarcoma U2OS cells via downregulation of the APEH–proteasome system, with the accumulation of the typical hallmarks of proteasome: NF-κB, p21^Waf1^, and polyubiquitinylated proteins. We found that the SsCEI 4 anti-proliferative effect involved a senescence-like growth arrest without noticeable cytotoxicity. These findings represent an important step toward understanding the mechanism(s) underlying the APEH-mediated downregulation of proteasome in order to design new molecules able to efficiently regulate the proteasome system for alternative therapeutic strategies.

## 1. Introduction

Osteosarcoma (OS) is the most common histological form of primary bone cancer and it is prevalent in children and young adults. OS tends to occur at the sites of bone growth, presumably because proliferation makes osteoblastic cells in this region prone to acquire mutations, for example in the retinoblastoma (RB) and *p53* genes, which could lead to cell transformation. Although chemotherapy has strongly contributed to reduce mortality, up to 50% of patients can develop chemoresistance. Therefore, much effort is being put into the development of new drugs targeting novel biologic pathways, whose regulation minimizes cross-resistance and increases response.

In this context, following the success in multiple myeloma (MM) treatment [1,2,3], proteasome inhibition and suppression of related resistance mechanisms have become an important focus in anticancer therapy [4,5,6]. Intracellular protein turnover regulates many fundamental processes in eukaryotic cells and the ubiquitin–proteasome system (UPS) plays a key role in these mechanisms since it represents the major non-lysosomal pathway through which proteins are degraded [7]. The 26S proteasome is a large intracellular protease (1500–2000 kDa) that is composed of two functional components: a 20S catalytic core and two 19S regulatory subunits [8]. The protease activity resides in a channel at the center of the 20S subunit, which harbors three enzymatic activities: chymotrypsin-like, trypsin-like, and post-glutamyl peptide hydrolase-like [8]. The main function of UPS is to protect the cells against the accumulation of misfolded, oxidized, or otherwise damaged and potentially toxic proteins, establishing a kind of cell quality control system [9,10]. Additionally, it also regulates the half-life of many proteins involved in important biological processes such as cell cycle, oncogenesis, or apoptosis [4,11], thereby playing a critical role in preserving cellular homeostasis. In order to sustain their high replication rate, which implies rapid protein synthesis and turnover, cancer cells are strongly dependent on the proper functions of UPS as compared to their normal counterpart [4,5]. Therefore, malignant cells are exceedingly vulnerable to proteasome inhibition, making the proteasome a therapeutic target in oncology and proteasome inhibitors (PIs) an important class of anti-cancer drugs [11,12,13]. Validation of this approach has been provided by Bortezomib, a highly selective and reversible inhibitor of the chymotrypsin-like activity of the proteasome complex [1,6]. Bortezomib is currently in use for the treatment of refractory multiple myeloma and mantle cell lymphoma [1,2,3,5]. Although PIs have achieved significant clinical benefit for the treatment of different types of tumors, toxic side effects and drug resistance limit their effectiveness. Therefore, in recent years, several studies have suggested that the targeting of functionally related proteasome effectors can be an alternative and safer way to recover proteasome dysfunction associated with pathological conditions [14].

Recently, we demonstrated that proteasome activity can be regulated through acylpeptide hydrolase (APEH)-mediated mechanisms in melanoma (A375) [15] and in differentiated adenocarcinoma cell lines (Caco-2) [16], thus representing a molecular target to indirectly control UPS functions and cancer cell proliferation. Specifically, by using a set of natural and synthetic APEH inhibitors, we showed that suppression of the APEH activity induces cell death without cytotoxicity by a mechanism involving the downstream inhibition of proteasome [15,16]. The strong correlation established between APEH and proteasome activities in a panel of cancer cell lines supported the idea that these enzymes are functionally interrelated [15]. APEH is one of the four members of the Prolyl OligoPeptidase class (POP, clan SC, family S9) [17]; it catalyzes the removal of *N*-acylated amino acids from acetylated peptides and it has been studied in a number of eukaryal [18], bacterial [19], and archaeal [20] organisms, although its physiological role has not been completely clarified. However, as previously suggested, APEH can be involved in the protein-degradation machinery, possibly acting as an antioxidant defense system [21,22,23,24,25], and could represent a promising therapeutic target for a wide array of diseases caused by misfolded protein accumulation [26,27].

In this work, we further investigated the functional correlation of APEH and proteasome at both transcriptional and activity levels in a panel of 13 human cancer cell lines and examined the effects of the APEH inhibitor SsCEI 4-treatment on cell viability. Specifically, we demonstrated that SsCEI 4 was able to significantly reduce the proliferation in osteosarcoma cells via downregulation of proteasome and by a cell death-independent mechanism.

Altogether, APEH targeting shows promise in the control of proteasome functions, opening new challenging perspectives for the improvements in cancer therapy.

## 2. Results and Discussion

### 2.1. SsCEI 4 Is Able to Inhibit Acylpeptide Hydrolase (APEH) Activity but Not Proteasome in Cell-Free Assays

APEH plays a relevant role in protein degradation processes and its activity is strictly interplayed with that of proteasome, contributing to cell growth and proliferation by a yet unknown mechanism [8,16]. On these bases, in the attempt to elucidate the functional relationship between APEH and proteasome, we investigated the impact of APEH down-regulation on the fate of cancer cells by using the recently identified APEH peptide inhibitor SsCEI 4, which is able to block the enzymatic activity in a competitive manner [16]. The IC_50_ value determined towards APEH in cell-free assays for SsCEI 4 was 84.0 ± 16.0 µM (Figure S1A) [16], while no effects were detected on the chymotrypsin-like (CT-like) activity associated with the β5 subunit of the proteasome purified from human erythrocytes, even at the highest concentrations tested (Figure S1B). In addition, the potent proteasome inhibitor Bortezomib (BTZ), which was used as a positive control (Figure S1C), was completely ineffective toward APEH (Figure S1A). Therefore, SsCEI 4 was used as a molecular tool to study the APEH-proteasome interrelation in cellular models.

### 2.2. mRNA, Activity and Protein Levels of APEH, and Proteasome Correlate in a Wide Panel of Cancer Cell Lines

A positive correlation was previously observed between APEH and proteasome activities in a small group of cancer cells, suggesting a tight functional interdependence [15,16]. To further investigate the mechanism of such molecular interplay, we measured the basal activity of these two enzymes and their relative expression and protein levels in a more extensive panel of cancer cell lines.

**Figure 1:**
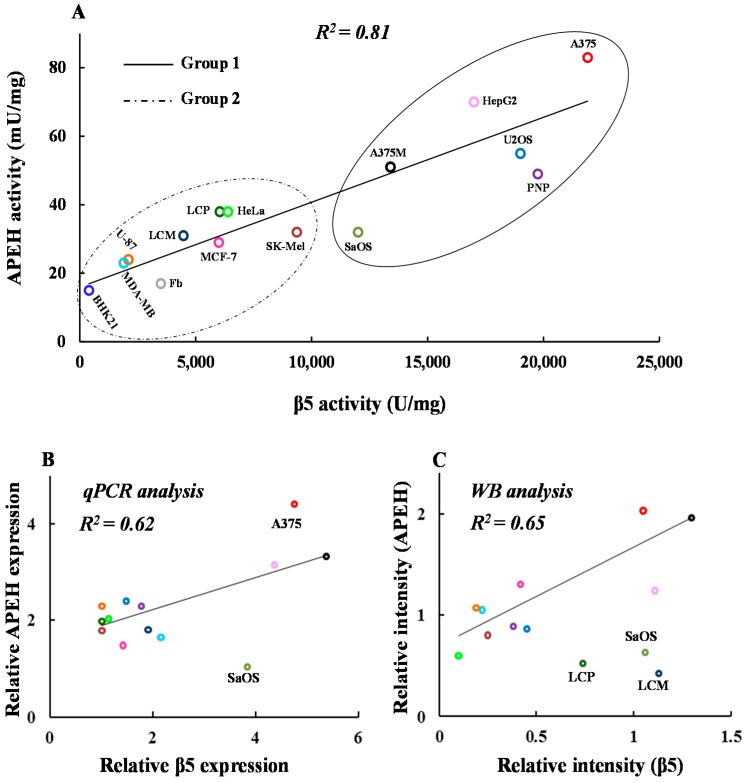
Activity, mRNA and protein levels of acylpeptide hydrolase (APEH), and proteasome correlate in a wide panel of cancer cells. Thirteen human cancer and two non-cancerous cell lines (BHK21, fibroblasts) were harvested at the pre-confluent stage and used for protein cytoplasmic or mRNA extract preparations. (**A**) Basal APEH and proteasomal CT-like (β5 subunit) specific activities were measured; (**B**) the mRNA levels of APEH and β5 proteasome subunit were evaluated by qPCR and reported as relative expression using the transcriptional levels in human fibroblast as control; (**C**) intracellular levels of β5 and APEH were detected by immunoblotting analysis. Data from three different analyses were normalized to the density of control protein (β-actin) and expressed as relative intensity. Results are presented as the mean values ± SD of triplicate analyses from at least three different experiments.

As shown in Figure 1A, a strong linear correlation between APEH and Chymotrypsin-like (CT-like) proteasome specific activities was still observed, supporting the hypothesis of a functional relationship between these two enzymes and evoking a not yet clarified physiological and/or pathological relevance. As can be seen, the high activities of both enzymes were associated with increased mRNA and/or protein amounts as revealed by the correlation between their relative mRNAs and protein levels in almost all cell lines (Figure 1B,C). Remarkably, U2OS (osteosarcoma cell lines) and PNP (malignant melanoma cell lines) displayed low levels of protein/RNA transcripts in spite of the high activity detected. In addition, by a more detailed examination of the scatter plot in Figure 1A, two distinct subsets of cell lines could be identified, one showing high (Group 1) and the other low (Group 2) APEH and proteasome activity levels.

In light of these results, we further investigated cells from Group 1, according to the hypothesis that these could be more sensitive to APEH and proteasome downregulation possibly due to their greater dependence on the function of this enzymatic system. It has been widely reported that malignant cells are susceptible to killing by proteasome inhibitors, as they are highly dependent on protein degradation processes due to a rapid proliferation compared to normal cells [3,6,28,29]. This behavior is especially evident in some types of cancer cells, such as those of multiple myeloma, which represent an important model system to study the anti-proliferative effects of proteasome inhibitors [1,4,5].

### 2.3. SsCEI 4 Is Effective in Reducing Osteosarcoma Cell Viability in a Dose- and Time-Dependent Manner

**Figure 2:**
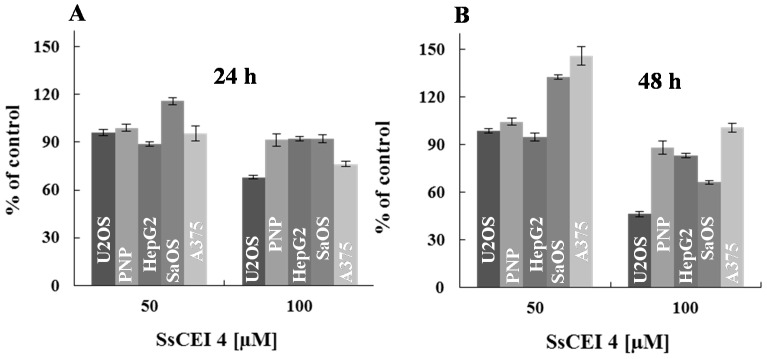
Human cancer cells exhibit differential sensitivity to the anti-proliferative activity of SsCEI 4. The effects of peptide on cell viability were assessed in cancer cell lines belonging to Group 1, exposed for 24 (**A**) and 48 h (**B**) to increasing concentrations of SsCEI 4. Data are normalized to the values obtained with the untreated cells and are expressed as means ± SD values of triplicate data from three independent experiments.

In the attempt to elucidate the role played by APEH in cellular proliferation, cells belonging to Group 1 were exposed to different doses of the APEH inhibitor SsCEI 4 (50 and 100 μM) and cell viability was evaluated after 24 and 48 h incubation. As reported in Figure 2A,B, SsCEI 4-treatment caused a marked dose- and time-dependent decrease in the viability of osteosarcoma cell lines (U2OS and SaOS) compared to untreated cells, reaching a maximal reduction of 63% and 40%, respectively, at the highest concentration used and at 48 h.

Interestingly, the ability of the peptide to markedly reduce the proliferation of osteosarcoma cells might indicate the existence of a tumour-specificity arising from peculiar features of these cell lines. Altogether, these data suggest that the high basal levels of proteasome and APEH might be a necessary but not sufficient condition to render cancer cells sensitive to APEH inhibition.

**Figure 3:**
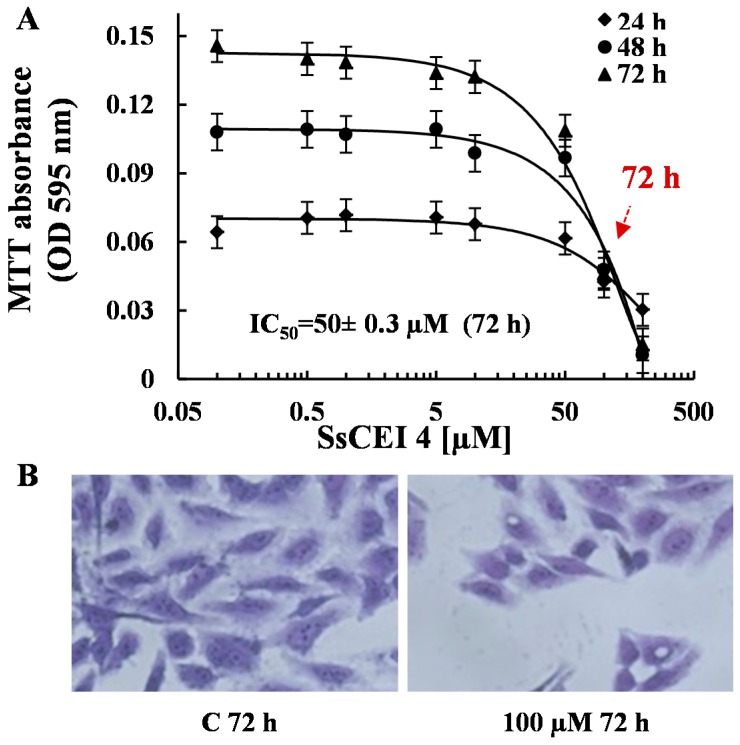
SsCEI 4 inhibits cell growth of U2OS cells in a dose- and time-dependent manner. U2OS cells were grown in the presence or absence of increasing concentrations of SsCEI 4 (0.1–200 µM) for 24, 48 or 72 h. (**A**) Inhibition of cell growth was assessed by 3-(4,5-dimethylthiazol-2-yl)-2,5-diphenyltetrazolium bromide (MTT) assay and values represent the means ± SD of triplicate data from three independent experiments; (**B**) microscope image (200×) of U2OS cells treated for 72 h with 100 µM SsCEI 4 and stained with crystal violet. Cells treated with DMSO were used as control (C).

Based on these results, we selected U2OS cells as the best responding model system to study the effects of APEH modulation of cell viability. Firstly, in order to define the dose accountable for 50% decrease of cell viability (IC_50_), U2OS cells were incubated for different times (24, 48, and 72 h) with increasing amounts of SsCEI 4 (ranging from 100 nM to 200 μM). The resulting isobolograms (Figure 3A) revealed that SsCEI 4 reduced the cell proliferation of U2OS in a concentration- and time-dependent manner, with a maximum observed at 200 μM at both 48 and 72 h treatment. The same effect was shown at 72 h by microscope image after staining with crystal violet (Figure 3B). The estimated IC_50_ was 50 μM at 72 h and 100 μM at 48 h, therefore 72 h was chosen as the time interval to set up further experiments. Proliferation data obtained using human fibroblasts as control supported the lack of toxic effects even at high concentrations of SsCEI 4 (data not shown).

### 2.4. SsCEI 4 Induces APEH-Proteasome Downregulation in U2OS Cells

**Figure 4:**
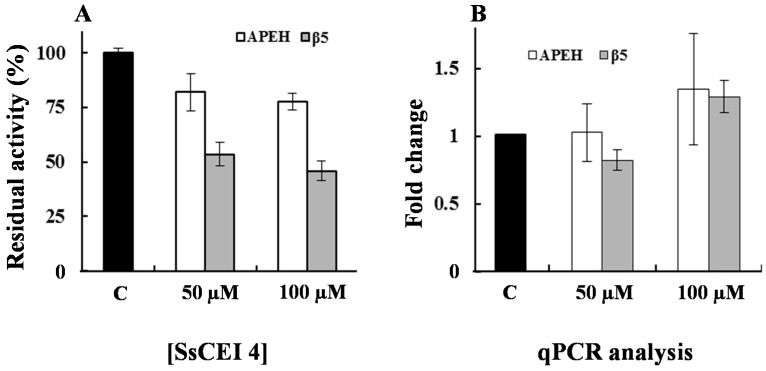
Anti-proliferative activity of SsCEI 4 correlates with the downregulation of APEH and proteasome in U2OS cells. (**A**) APEH (white bars) and CT-like proteasome (gray bars) activities were measured in pre-confluent U2OS cells incubated with 50 and 100 µM SsCEI 4 for 72 h. Untreated cells (C) were used as controls. Measurements of APEH or proteasomal CT-like activities were performed on cytoplasmic extracts; (**B**) The mRNA levels of APEH and β5 proteasome subunit were evaluated by qPCR and expressed as fold change in comparison to untreated cells (C). Results are presented as the mean values ± SD of triplicate analyses from at least three different experiments.

To verify whether proteasome activity was decreased in U2OS cells following APEH inhibition, cells were incubated with 50 or 100 μM SsCEI 4 for 72 h and protein extracts were assayed for enzyme activities. As shown in Figure 4A, APEH activity was dose-dependently reduced (about 25%) in the presence of the peptide inhibitor and this trend was paralleled by a marked reduction of CT-like (β5 subunit) proteasome activity (54%), which reached its maximum effect at 100 µM. To gain insights into the mechanism underlying the SsCEI 4 anti-proliferative effects, we performed gene expression analysis on cell extracts. As shown in Figure 4B, a slight increase (about 25%) of the transcriptional levels of the two genes was observed with the highest dose of the inhibitor, probably due to the need of cells to compensate for the reduction of the two enzymatic activities. However, as the treatment did not cause a strong variation of APEH and β5 gene transcripts, the APEH and proteasome modulation induced by SsCEI 4 was not at transcriptional level.

Therefore, proteasome deregulation occurs via a more complex pathway triggered by APEH inhibition, since SsCEI 4 is not able to affect directly proteasome function (Figure S1B) and that its anti-proliferative effect involves a specific down-regulation of the APEH-proteasome system. Indeed, to support the cooperative role for the APEH–proteasome system in the control of protein turnover, we previously observed a marked accumulation of a protein misfolded model (CFTR-M) typically degraded by UPS, following the transfection of CFBE41o-DF cells with APEH siRNA [16].

This suggestion was corroborated by evaluating the cytoplasmic levels of some canonical hallmarks of proteasome inhibition, such as NF-κB, p21^Waf1/Cip1^, and polyubiquitinylated proteins.

**Figure 5:**
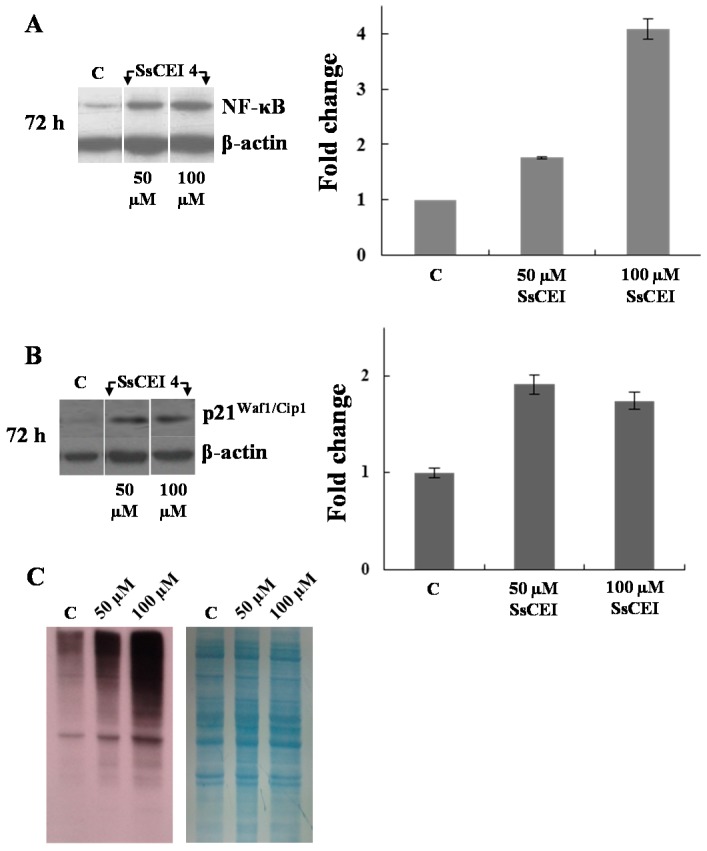
SsCEI 4 treatment of U2OS induces an increase of the classical hallmarks of the proteasome inhibition. Representative immunoblots of NF-κB (**A**) and p21^Waf1/Cip1^ (**B**) in U2OS cells exposed to SsCEI 4 (50 and 100 µM) for the indicated time intervals. Data were normalized to the density of control protein (β-actin). The values were expressed as average relative intensity as compared to untreated cells (C) and expressed as means ± SD of measurements performed in triplicate; (**C**) Polyubiquitinated proteins in U2OS cells untreated (C) and treated with SsCEI 4 for 72 h. After immunodetection, the polyvinylidene difluoride (PVDF) membrane was stained with Coomassie blue.

As shown in Figure 5A, treatment of U2OS with different amounts of SsCEI 4 for the indicated times, resulted in a dose- and time-dependent increase of NF-κB levels compared to control cells. A comparable accumulation of the cyclin-dependent kinase inhibitor p21^Waf1/Cip1^ was observed after SsCEI 4 treatment (Figure 5B), together with a dose-dependent increase of the levels of high molecular-mass anti-ubiquitin immunoreactive species (66 to 160 kDa, Figure 5C), indicative of polyubiquitin conjugates.

**Figure 6:**
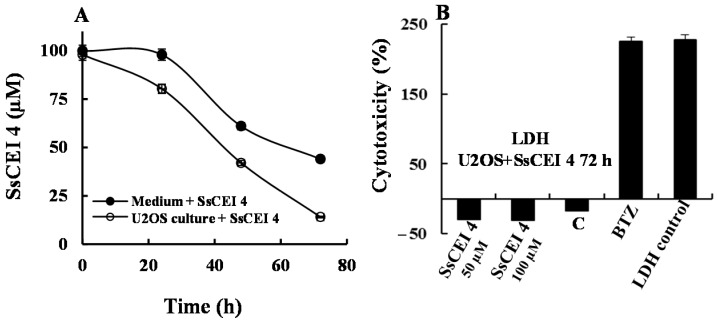
SsCEI 4 treatment of U2OS does not induce cytotoxicity. (**A**) Residual amount of SsCEI 4 detected in Dulbecco’s Modified Eagle’s medium (DMEM) alone (black circle) and in DMEM in contact with U2OS cancer cells (white circle) at 24, 48 and 72 h. Experiments were performed using peptide solutions at 100 µM; the residual peptide amount was determined by reverse-phase High-performance liquid chromatography (RP-HPLC) using the pure peptide as standard. Data are representative of at least two independent experiments; (**B**) Cytotoxic effects of SsCEI 4 at two different concentrations on U2OS cells was evaluated by measuring the LDH release in the culture medium. U2OS cells, treated with DMSO (C) or with BTZ (10 nM) were used as controls. U2OS cells were treated with cell lysis buffer to induce maximum LDH leakage (LDH control). Lactate Dehydrogenase (LDH) activity was determined by a fluorescent assay (*n* = 3). Data are reported as percentage of maximum LDH release and values are presented as means ± SD.

Finally, we determined the average fraction of SsCEI 4 that, surviving degradation by medium proteases or escaping the capture by serum proteins (mostly albumin), crossed U2OS membranes to reach its specific target. Data showed that at 72 h treatment about 60% of the peptide added to the medium was in some way subtracted in the presence of serum, but a fraction of about 30% likely entered cells and expectedly fulfilled APEH inhibition (Figure 6A). Therefore, the actual peptide IC_50_ value (50 μM) of these cells should be lower than that determined by the MTT assay (Figure 3A) and corrected in consideration of this crucial aspect.

### 2.5. SsCEI 4 Treatment of U2OS Does Not Induce Cytotoxicity, Apoptosis, or Autophagy

To elucidate the molecular mechanism underpinning the reduced cell proliferation triggered by SsCEI 4, cytotoxicity, apoptosis, and autophagy biomarkers were investigated. Firstly, LDH activity was evaluated in culture medium following 72 h exposure to 50 and 100 μM SsCEI 4, to exclude a non-specific cytotoxic effect of the peptide on cells. As shown in Figure 6B, a strong cytotoxicity (exceeding 200%) resulted from Bortezomib (BTZ, 10 nM) supplementation used as positive control, while LDH activity in cell cultures treated with SsCEI 4 at both concentrations was comparable to that of DMSO-exposed cells, indicating that the suppression of cell viability induced by SsCEI 4 was not associated with any cytotoxic effect. It is widely reported that activation of executioner caspases is a major hallmark of apoptotic cell death [30]. Therefore, to assess whether SsCEI 4 induced caspase 3 activation in U2OS cells, western blot analysis was performed on whole cell lysates collected at 72 h after treatment with 50 and 100 µM SsCEI 4. Using an antibody that specifically recognizes both the inactive (pro-caspase 3) and the cleaved “activated” forms of caspase 3, no activation was observed after treatment (Figure S2A). This was further verified performing an in vitro fluorogenic assay using the substrate Ac-DEVD-AMC (Figure S2B). Similar results were observed looking at the cleavage of poly(ADP-ribose)polymerase (PARP-1), a 113 kDa nuclear enzyme that is fragmented during apoptosis, as confirmed by using Etoposide (ETP) as a positive control (Figure S2C) [31]. In the same cells, activation of SsCEI 4-induced autophagic pathways were biochemically analyzed by measuring the levels of Beclin-1 and LC3-I/LC3-II proteins, which accumulate during autophagy. As shown in Figure S3, Western blot analyses performed on whole cell lysates of U2OS cells collected at 72 h after treatment with 50 and 100 μM ScCEI 4 showed no increase in Beclin-1 levels nor in LC3-II, the cleaved form of LC3-I (Figure S3B), which were instead strongly induced by Quercetin (Q), a potent autophagy inducer used as a positive control [32]. Altogether, our results suggest that SsCEI 4 does not suppress U2OS cell viability via canonical cytotoxicity, apoptosis, or autophagy mechanisms.

### 2.6. SsCEI 4 Treatment Promotes Senescence and Alteration of Cell Cycle Regulators in U2OS Cells

The cell cycle is a highly regulated event in which successive protein synthesis, activation, and deactivation occur. Progression through each phase is controlled by a co-operative activity of distinct cyclin-dependent kinases (CDKs) and their regulatory subunits, cyclins, which are specific for different CDK subunits [33]. The kinase activities of the CDK-cyclin complexes are negatively modulated by CDK inhibitors (CKIs) including p16^INK4a^, p21^Waf1/Cip1^, and p27^Kip1^, all of which modulate both cell proliferation and cell death. Specifically, p16^INK4a^ is a well characterized inhibitor of d-type cyclin-dependent kinases [34] and its upregulation is one of the pivotal nodes of activated tumour suppressor network during senescence [35,36], arresting cells in early G1 phase. In this complex pathway, the proteasome system covers a crucial role in cell cycle progression because it controls the proteolytic degradation of cyclins and CKIs [37,38,39,40].

**Figure 7:**
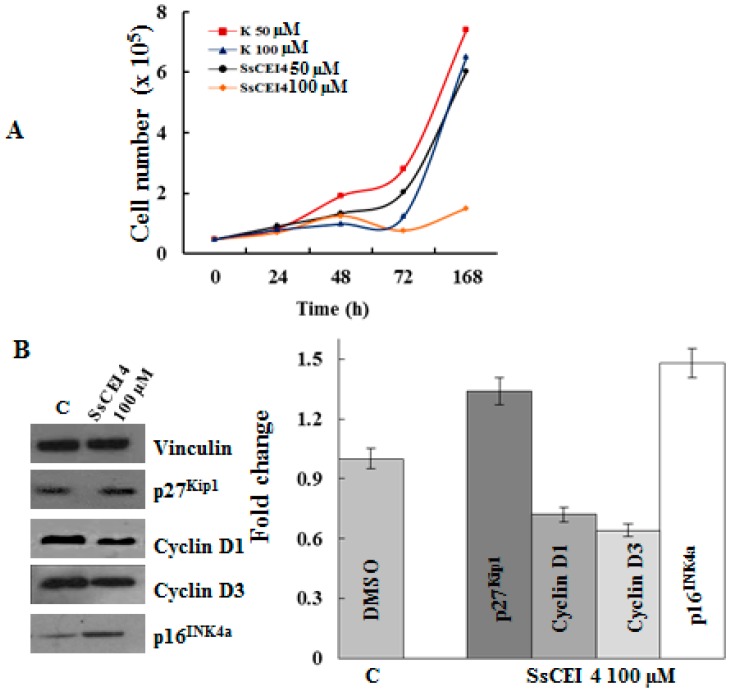
Expression of cell cycle regulators in U2OS cells following SsCEI 4 treatment. (**A**) U2OS cells (1 × 10^4^/cm^2^) were treated with vehicle (C) or various concentrations of SsCEI 4 (50 and 100 µM) for 24, 48, 72, and 168 h. Cell numbers at each time point were determined by trypan blue exclusion dye; (**B**) Cell lysates were prepared from U2OS following treatment with vehicle DMSO (C) or 100 µM SsCEI 4 for 72 h. Equal amounts of whole cell lysates were subjected to electrophoresis and analyzed by Western blot for cyclin D1, D3, p16^INK4a^, and p27^Kip1^. Vinculin was used as a loading control. Results of densitometry bands are expressed as the mean of ratio between densitometries of each regulator and Vinculin bands. Data are representative of two experiments performed in triplicate.

Since SsCEI 4 caused a dose-dependent reduction of U2OS cell growth, without a net increase in the cell number at 100 µM concentration (Figure 7A), we examined the expression changes of different key cell-cycle regulators and senescence-associated proteins, such as p16^INK4a^, p27^Kip1^ and D cyclins. As shown in Figure 7B, cyclin D1 and cyclin D3 expression significantly reduced in response to peptide treatment and in parallel, upregulation of CKIs p16^INK4a^ and p27^Kip1^ (Figure 7B) was also evidenced. These effects were observed both at 50 µM (data not shown) and 100 µM of SsCEI 4 suggesting a dose-dependent response of these regulators. Altogether, these data suggest that the peptide inhibitor is able to promote senescence thought cell cycle arrest at the G1 phase in U2OS. Specifically, the observed decrease of cyclin D1 and D3 levels could account for the block at G1 because their up-regulation is the key step governing the exit from the G1 phase and progression in the next cell cycle phase [33]. This is in line with a marked increase in the protein levels of the typical biomarkers of senescence, p16^INK4a^, p27^Kip1^ (Figure 7B) [41], and p21^Waf1/Cip1^ (Figure 5B).

In conclusion, SsCEI 4 may contribute to the induction and/or maintenance of a non-proliferative state of U2OS cells, possibly through an APEH-mediated inhibition of UPS, which consequently may promote a senescence-like growth arrest in these cell lines.

## 3. Materials and Methods

### 3.1. Reagents

Porcine liver APEH was purchased from Takara BIO INC. (Shiga, Japan) and 20S human proteasome from Boston Biochem (Boston, MA, USA). Bortezomib (BTZ) was obtained from Santa Cruz Biotechnology (Heidelberg, Germany). The chromogenic substrate acetyl-alanine-*p*-nitroanilide (Ac-Ala-pNA) and the fluorogenic substrate *N*-succinyl-Leu-Leu-Val-Tyr-7-amido-4-methylcoumarin (Suc-LLVY-AMC) were purchased from Sigma-Aldrich (Milan, Italy). All chemicals of the highest purity were from Sigma-Aldrich (Milan, Italy) or Calbiochem (Milan, Italy).

The following antibodies were used: anti-APEH antibody (sc-102311, Santa Cruz Biotechnology); anti-β-actin (sc-47778, Santa Cruz Biotechnology); Vinculin (ORB 76294, Biorbyt, Berkeley, CA, USA); anti-NF-κB/p65 antibody (RB-1638-PO, Thermo Scientific, Waltham, MA, USA); anti-p21Waf1 antibody (11-338-C100, Exbio, Prague, Czech Republic); anti-caspase 3 (sc-7148, Santa Cruz Biotechnology); anti-β5 subunit proteasome 20S (BML-PW8895, Enzo Life Sciences, Farmingdale, NY, USA); anti-poly(ADP-ribose) polymerase (PARP, ab6079, Abcam, Cambridge, UK); anti-LC3 and anti-Beclin-1 (4445S, Autophagy Antibody Sampler Kit, Cell Signaling Technology, Danvers, MA, USA); anti-mono- and polyubiquitinylated conjugates (FK2) (horseradish peroxidase HRP conjugated) (BML-PW0150, Enzo Life Science); cyclin D1, D3 and p27Kip1 (9932, Cell Cycle Regulation Sampler Kit, Cell Signaling Technology); p16^INK4a^ (sc-759, Santa Cruz Biotechnology).

### 3.2. Enzyme Assays

APEH activity was measured spectrophotometrically using the chromogenic substrate Ac-Ala-pNA (Bachem, Laufelfingen, Switzerland). The reaction mixture (1 mL) containing pure APEH (38 ng) or an appropriate amount of cell extract, was preincubated at 37 °C in 50 mM Tris-HCl buffer pH 7.5 (Tris Buffer) for 2 min. Then, 1 mM Ac-Ala-pNA was added and the release of *p*-nitroaniline (ε410 nm = 8800 M^−1^·cm^−1^) was measured by recording the absorbance increase at 410 nm on a Cary 100 Scan (Varian) UV/Vis spectrophotometer, equipped with a thermostated cuvette compartment. APEH activity was expressed in IU. The synthetic fluorescent substrate Suc-LLVY-AMC was used for the measurement of the chymotrypsin-like (CT-like) activity of proteasome, at a final concentration of 0.080 mM. The reaction mixture (0.9 mL) containing appropriate amounts of proteasome was preincubated in Tris buffer. Suc-LLVY-AMC was added, and the release of the fluorescent product (7-AMC) was monitored for 5 min in a Perkin–Elmer LS 50 B fluorimeter. The excitation and emission wavelengths were 380 and 460 nm, respectively. Protease inhibitor activities of the peptide SsCEI 4 and Bortezomib were carried out using fixed amounts of commercially available APEH or 20S proteasome and increasing peptide concentrations. Mixtures were pre-incubated for 30 min at 37 °C before addition of the substrate, and the enzymatic activities were followed as described above. The IC_50_ (concentration required for obtaining 50% of the maximum effect measured) values were determined.

### 3.3. Cells, Culture Conditions and Treatments

Human cancer cells were grown in DMEM (Gibco, Milan, Italy) supplemented with 10% FBS (Fetal bovine serum), 2 mM l-glutamine in a humidified atmosphere at 37 °C in 5% CO_2_. LCM (melanoma cells from metastatic lymph nodes), LCP (cells from primary melanoma), PNP (malignant melanoma cell lines), and SK-Mel (human melanoma cell lines) were kindly donated by Giuseppe Palmieri (ICB-CNR). Other cell lines were obtained by ATCC (LCG Standards, Milan, Italy). Cells were routinely counted manually with a hemocytometer using trypan blue dye exclusion staining. SsCEI 4 peptide was dissolved in dimethyl sulphoxide (DMSO, Carlo Erba, Milan, Italy). Cells were treated with peptide and control cells were exposed to the same amount of vehicle.

### 3.4. Protein Extraction and Western Blotting Analyses

Following peptide or control treatments, cells were washed three times with ice cold phosphate-buffered saline and collected immediately at 4 °C in lysis buffer (0.5% Nonidet P40, 25 mM Hepes, pH 7.5, 100 mM NaCl and complete protease inhibitors (Roche, Basilea, Switzerland). Lysates were centrifuged at 13,000*× g* for 15 min at 4 °C. The protein concentration was determined in supernatants by the Bradford assay method [42] before their use in enzymatic assays or SDS-PAGE (Sodium Dodecyl Sulphate-PolyAcrylamide Gel Electrophoresis) [43].

For Western blotting analysis, protein aliquots (30 µg/lane) were run on SDS-PAGE (8% or 12.5%) and then electroblotted onto PVDF membranes (ImmobilonTM, Millipore, Milan, Italy). Membranes were next incubated with the specific primary antibodies (1 h at room temperature) and then incubated with the appropriate dilution of secondary antibodies (1 h at room temperature). Immunocomplexes were visualized by enhanced chemiluminescence and autoradiography according to the manufacturer protocol (Santa Cruz Biotechnology) and quantified by densitometric analysis with Image J software (NIH, Bethesda, MA, USA), using the β-actin and Vinculin levels as loading control for normalization.

### 3.5. MTT Assay and Crystal Violet Staining

The colorimetric 3-(4,5-Dimethyl-2-thiazolyl)-2,5-diphenyl-2*H*-tetrazolium bromide (MTT) (Sigma-Aldrich, Milan, Italy) assay was performed to assess cell viability. Briefly, cells were seeded in 50 µL medium per well in 96-well plates and incubated overnight to allow adhesion. Subsequently, cells were incubated for 24, 48 and 72 h in the presence of different concentrations of peptide. After the treatment the medium was removed and the cells were incubated with MTT (0.5 mg/mL) in DMEM *w*/*o* (without) red phenol for 3 h at 37 °C. Then cell supernatants were discarded and isopropanol plus 0.1 N HCl was added to dissolve MTT crystals. The absorbance was detected at 595 nm using a microplate reader (BioRad Model 680 Microplate reader). The relative number of viable cells was expressed as a percentage of the control. Microscopy analysis of the cells treated with SsCEI 4 for 72 h was performed with crystal violet. Briefly, the culture medium was removed and the cells were fixed with 10% formalin solution (Sigma-Aldrich) for 15 min at dark and stained with 0.1% *v*/*w* crystal violet (Sigma-Aldrich); after 30 min, the cells were washed twice with double distilled water and left to dry. The U2OS cells were visualized and digital photos were taken with Zeiss Axiovision microscope (Carl Zeiss Microscope Inc., Jena, Germany).

### 3.6. Apoptosis Assays

To assess apoptosis, cleavage of PARP and caspase 3 was determined by Western blot in U2OS cells treated with 50 and 100 μM of SsCEI 4 for 72 h. Jurkat cells treated with 25 µM Etoposide (ETP) were used as a positive control. The caspase 3 activity was measured as previously reported in Palmieri et al. [16].

### 3.7. Cytotoxicity Assay

The release of LDH (Lactate dehydrogenase) was used as a marker for cell toxicity. After treatment with 50 and 100 µM SsCEI 4 for 24 h, cleared supernatants were incubated with reaction buffer for 30 min at room temperature according to the manufacturer instructions (ab65393, LDH-Cytotoxicity Assay Kit II). Absorbance was measured at 490 nm and the cytotoxicity percentage was calculated as: [(Absorbancetest sample − Absorbancelow control)/(Absorbancehigh control − Absorbancelow control) × 100].

### 3.8. Cellular Internalization Assay of SsCEI 4 Peptide

The U2OS cells were treated with 100 µM SsCEI 4 for 72 h. Then, the media were collected, filtered through a Microcon YM filter (Millipore) with a cutoff of 10,000 Da at 4 °C and centrifuged at 13,000*× g*. The eluate recovered after each filtration (0.18 mL) was analyzed by reverse-phase HPLC (BioLC; Dionex, Thermo Scientific, USA) on a µBondapak C18 column (300 × 3.9 mm ID; Waters, Milford, MA, USA) eluted with a linear gradient of acetonitrile 0.1% TFA from 5% to 95% in 37 min at a flow rate of 1 mL/min. For each assay, culture media containing only SsCEI 4 (100 µM) were run in parallel and used as control samples. Peak area was used to calculate the relative amount of peptide internalization in cells.

### 3.9. Quantitative Real-Time PCR (qPCR) Analysis

Total RNAs from U2OS cells were isolated according to the procedure described in “High Pure RNA Isolation Kit” Protocol Handbook (Roche). RNA concentrations were determined with a Qubit Fluorometer (Invitrogen). RNAs were then reverse transcribed with the SuperScript VILO MasterMix (Invitrogen, Carlsbad, CA, USA). An amount of 100 ng of cDNA and its dilution series to calculate the efficiency of primers were used as template for quantitative qPCR amplifications. The assays were performed on an iCycler iQTM (Bio-Rad, Hercules, CA, USA) with 300 nM gene-specific primers, iTaqTM Universal SYBR^®^ Green Supermix (Bio-Rad, Hercules, CA, USA), and the following PCR conditions: one cycle at 95 °C for 10 min, 40 cycles at 95 °C for 15 s, 60 °C for 30 s, and 72 °C for 30 s. The expression level of the β-actin gene was used as an internal control for normalization. Raw Cycle threshold values (*C*_t_ values) obtained for the target genes were compared with the *C*_t_ value obtained for the β-actin gene (reference gene). All data were expressed as mean expression fold from triplicates and the final graphical data were derived from the following equation: *R* = (Etarget)Δ*C*_t__target (control − sample)/(Eref)Δ*C*_t__ref (control − sample) [44]. The Universal Probe Library Assay Design Center was used for the design of primers [45]. The primers used in the assay are listed below: β-actin dir 5′-CCAACCGCGAGAAGATGA-3′; β-actin rev 5′-CCAGAGGCGTACAGGGATAG-3′; APEH dir 5′-CCCCATTCATCCTTTGTCAC-3′; APEH rev 5′-AAAGCCCATCTTGCAAAGC-3′; β5 dir 5’-CATGGGCACCATGATCTGT-3’; β5 rev 5’-GAAATCCGGTTCCCTTCACT-3’; Beclin-1dir 5’-AGTACCTGAACCGGCACCT-3’; Beclin-1rev 5’-GCCGTACAGTTCCACAAAGG-3’; LC3dir 5′-CGCACCTTCGAACAAAGAG-3′; LC3rev 5′-CTCACCCTTGTATCGTTCTATTATCA-3′.

### 3.10. Statistical Analysis

Data were from three independent experiments performed in triplicate and means values (± Standard Deviation (S.D.)) were reported. Statistical analysis and IC_50_ calculation were assessed using the SigmaPlot 10.0 software [15].

## 4. Conclusions

Proteasome is a ubiquitous enzymatic complex that modulates protein homeostasis and controls the turnover of regulatory proteins involved in critical cellular processes including cell cycle progression and apoptosis [7]. It has been widely reported that human cancer cells are more sensitive to proteasome inhibitors than normal cells, indicating that proteasome knockdown represents a major approach to regulate tumour cell proliferation [1,4,5,6]. Hence, in the last decade, several proteasome inhibitors have reached the market with indications in otherwise incurable diseases such as multiple myeloma, but their administration requires a rigorous control to restrict the life-threatening side effects [1,2,3,4]. In the search of alternative anti-tumoral therapeutic targets, we have previously identified APEH as an enzyme that is functionally correlated to proteasome and is able to negatively modulate its activity through a yet unknown mechanism [15,16]. Therefore, in a scenario where both enzymes are ubiquitously present in healthy and diseased tissues [46,47,48,49,50], targeting of APEH appears to be a very interesting and novel approach to suppress proteasome activity in cells or tissues. In this study we have identified in U2OS, an osteosarcoma-derived cancer cell line, a suitable system model to elucidate the molecular events underlying APEH-proteasome interrelationship. Indeed, U2OS-treatment with the specific APEH inhibitor SsCEI 4 has induced a strong anti-proliferative effect associated to a concomitant down-regulation of both APEH and proteasome. These data are suggestive of a mechanism whereby APEH may indirectly control the proteasome activity level, as evidenced by the lack of any direct suppression of proteasome activity by SsCEI 4 or APEH-mediated proteasome degradation (Gianna Palmieri, unpublished results). In this regard, the hypothesis that APEH impairs the proteasome function by uncovering the N-tail of a yet unknown effector or by a negative feedback mechanism due to the accumulation of *N*-acetylated peptide products generated by proteasome-mediated protein degradation, cannot be ruled out.

Osteosarcoma is a life-threatening disease that affects the bones. The five-year survival rate is in the range between 60% and 80% in non-metastatic tumors, while it falls to about 15% to 30% in metastatic cases. Treatments include surgery and chemotherapy with methotrexate and doxorubicin among the others (www.cancer.org), thus no specific drugs have been developed for this tumour so far. In this context, the understanding of the molecular mechanisms responsible for the reduced U2OS proliferation triggered by APEH inhibition may have overwhelming implications for the development of more specific and focused osteosarcoma treatments. Therefore, we have deeply investigated how viability of these cells was influenced by SsCEI 4 and whether they affected cell death, cell proliferation rates or both. Notably, we have found that, despite all markers of proteasome suppression were largely increased, major cell death mechanisms, such as apoptosis and autophagy, were not activated. Instead, the induction of a non-proliferative state of U2OS cells can be consequent to a senescence-like growth arrest, as demonstrated by an impairment of the important cell cycle regulators (p27^Kip1^, p16^INK4a^; D1 and D3 cyclins). These observations are in contrast with those reported for the specific proteasome inhibitors that induce strong and sustained cell death [1,6,13]. Hence, a more complex mechanism might account for the substantial decrease of U2OS cell proliferation triggered by APEH inhibition, an event that may affect the cellular “fitness,” rendering the cells more susceptible to death-inducing activity of therapeutic drugs. To date, the elucidation of such pathways is yet to be defined but it may have strong implications for developing combination therapy to overcome drug resistance in osteosarcoma as well as in other cancer cells, which are highly dependent on APEH and proteasome function.

## Supplementary Materials

Supplementary materials can be found at www.mdpi.com/1422-0067/17/10/1614/s1.
