# Supplementary material for: APEH Inhibition Affects Osteosarcoma Cell Viability via Downregulation of the Proteasome
Source: Int J Mol Sci. 2016 Sep 23;17(10):1614. doi: 10.3390/ijms17101614 (PMC5085647; doi:10.3390/ijms17101614)
Supplement: Supplementary file 1 [file ijms-17-01614-s001.pdf]

Supplementary Materials: APEH Inhibition Affects Osteosarcoma Cell Viability via Downregulation of the Proteasome

Rosanna Palumbo, Marta Gogliettino, Ennio Cocca, Roberta Iannitti, Annamaria Sandomenico, Menotti Ruvo, Marco Balestrieri, Mosè Rossi and Gianna Palmieri

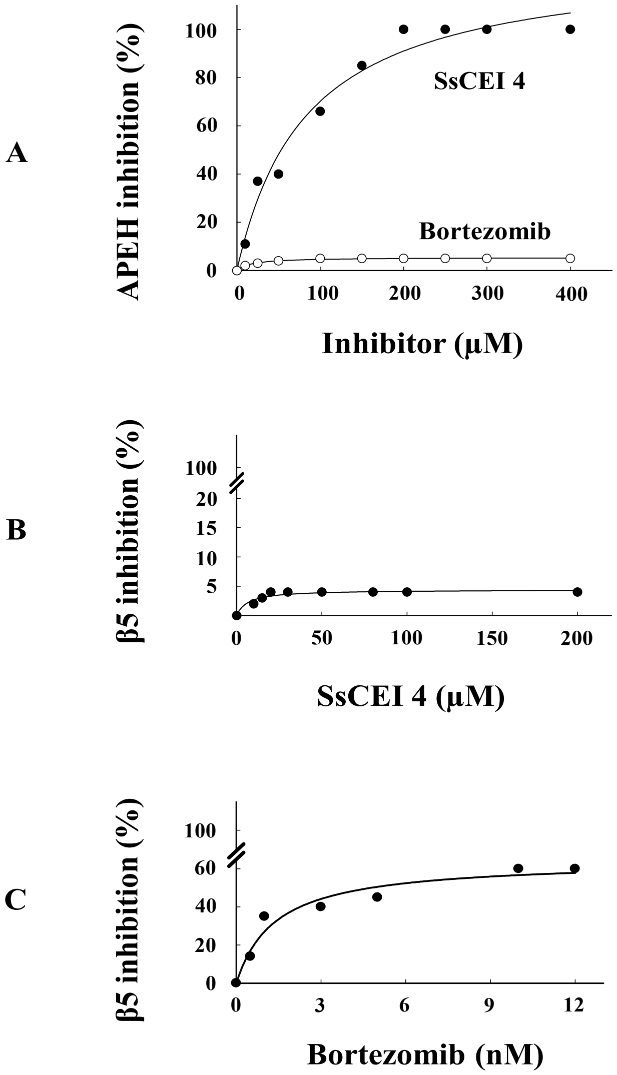

Figure S1. Inhibition curves of SCEI 4 and Bortezomib towards APEH and chymotrypsin-like (CT-like) subunit (β5) of proteasome; (**A**) Inhibition kinetic analyses of SCEI 4 and Bortezomib towards porcine APEH, using Ac-Ala-pNA as substrate; (**B**) inhibition kinetics by increasing concentrations of SsCEI 4 towards the CT-like (β5 subunit) activity of proteasome, using Suc-LLVY-AMC as substrate; (**C**) dose-dependent inhibitory effect of Bortezomib on CT-like activity of proteasome. Enzymes incubated without inhibitors were used as controls. The commercially available 20S proteasome from human erythrocytes and APEH from porcine liver were used to detect the inhibitory effects of the different compounds. The hyperbolic curves indicate the best fits for the data obtained, with IC_{50} values calculated from the graphs by SigmaPlot 10.0 software. Results are presented as the mean ± standard deviation (SD) of analyses from three independent experiments. SD values lower than 5% were not shown.

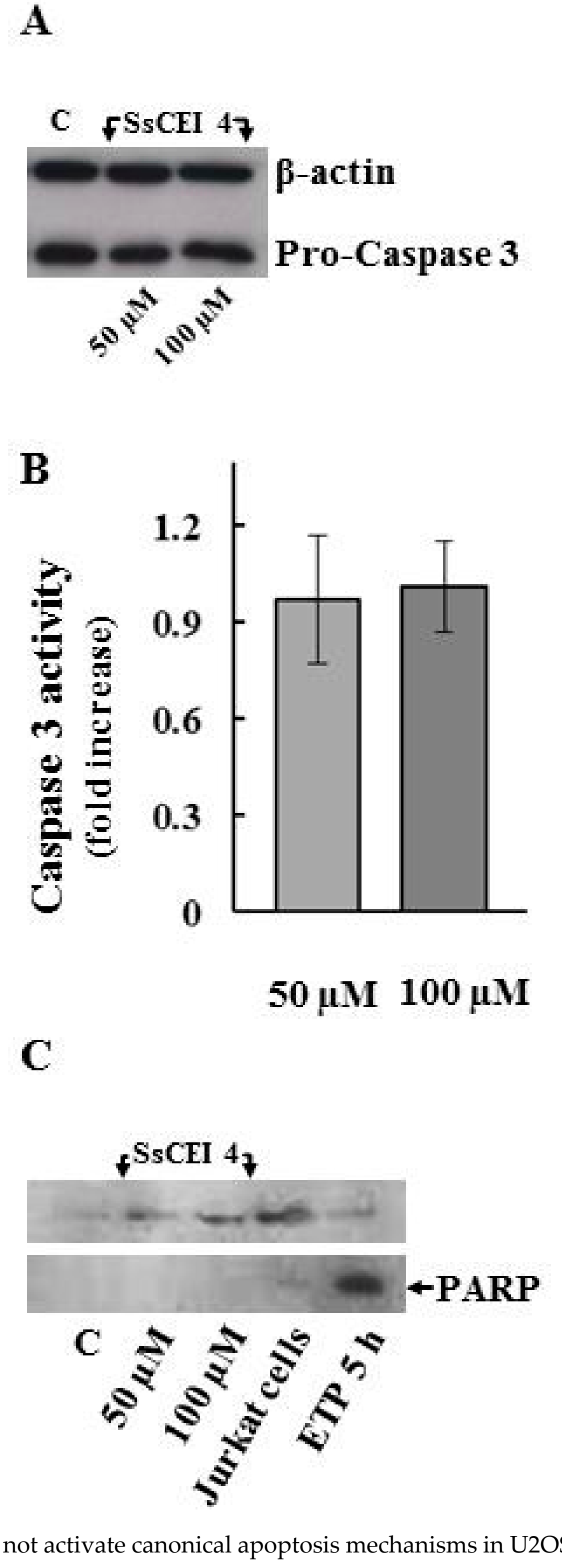

Figure S2. SsCEI 4 does not activate canonical apoptosis mechanisms in U2OS cells. (**A**) Lysates from U2OS cells, treated with vehicle DMSO (C) or with increasing concentrations of SsCEI 4 (50 and 100 µM) for 72 h, were analyzed by Western blot. Apoptosis was determined by detection of caspase 3 activation using a specific antibody raised against the inactive pro–caspase 3 (full length) and active cleaved fragments (caspase 3); (**B**) Caspase 3 activity was measured in cytoplasmic extracts obtained from U2OS cells treated for 72 h with 50 or 100 µM SsCEI 4 using the fluorogenic substrate Ac-DEVD-AMC and expressed as fold increase in comparison to untreated cells; (**C**) Western blot analysis of PARP-1 cleavage on lysates collected from U2OS cells exposed for 72 h to DMSO (C) or to 50 and 100 µM SsCEI 4. Jurkat cells treated with Etoposide (ETP) for 5 h were used as positive control and β-actin as loading control. Data are representative of at least three independent experiments.

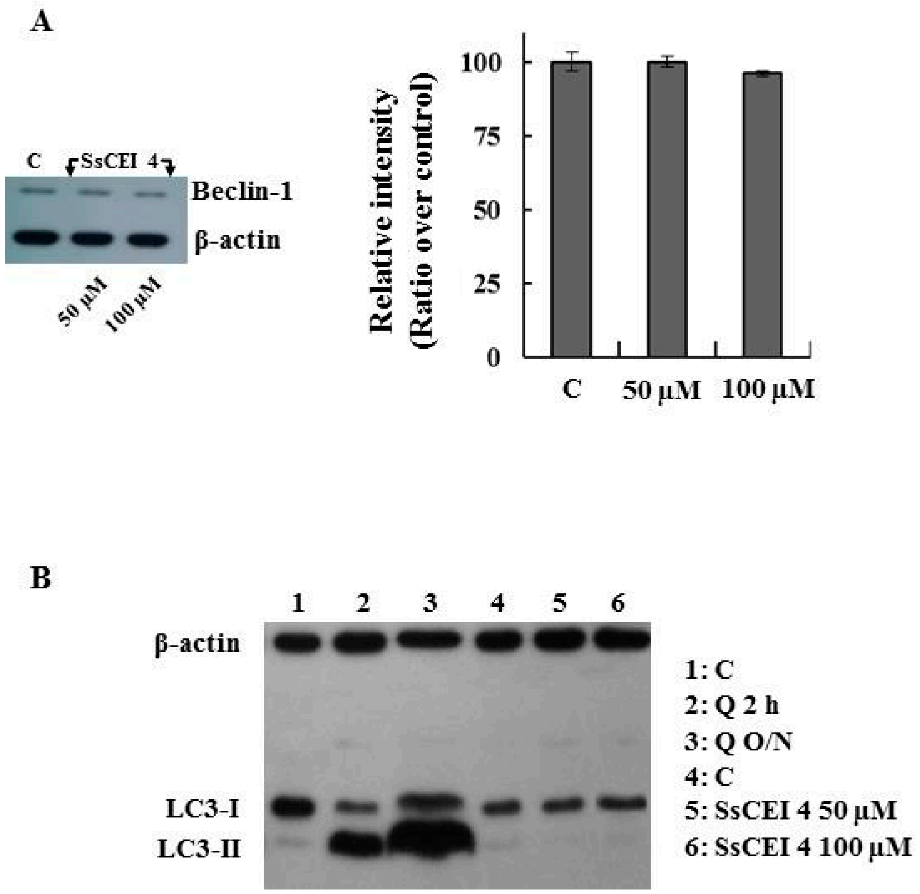

Figure S3. SsCEI 4 does not activate canonical autophagy mechanisms in U2OS cells. (**A**, *left panel*) U2OS cells were treated with DMSO (C) or SsCEI 4 (50 and 100 µM) for 72 h. Then, cell lysates were harvested and subjected to immunoblotting for Beclin-1; (**A**, *right panel*) Results of densitometry of Beclin-1 bands are expressed as the mean of relationships between densitometries of Beclin-1 and β-actin bands, used as loading control; (**B**) U2OS cells were treated with DMSO (C) or SsCEI 4 (50 and 100 µM) for 72 h, and cell lysates were used to analyze LC3-II conversion by immunoblotting using the specific antibody against LC3, which is able to recognize both the upper LC3-I band and the lower LC3-II. Cells treated with Quercetin (Q) for 2 h and overnight (O/N) were used as a positive control. Equal loading was confirmed by blotting with an anti-β-actin antibody. The images are representative of three independent experiments.
